# The Hygiene and Treatment of Catarrh

**Published:** 1881-04

**Authors:** 


### BIBLIOGRAPHICAL.

The Hygiene and Tteatment of Catarrh. Part I—Hygiene and Treatment. Part II—Thereapeutic Measures. With forty illustrations. By Thos. F. Rumbold, M.D. Geo. D. Rumbold & Co., publishers, St. Louis. 1881.

The disease catarrh, besides being very prevalent, is conceded to be very obstinate in resisting efforts made to affect its cure. We have received many works recently on this topic, and each author claims more or less success in the treatment of the dreaded disease. One and all agree in one essential requirement that must be regarded would we be successful in the treatment of catarrh, namely: the observance of certain general hygienic considerations, and a continued and even prolonged application of the proper thereapeutical measures.

The above work advocates this hygenic and thereapeutical persistency, and we commend it to those who desire to read up on catarrh.

